# 42 The Overt Pulmonary and Systemic Pro-inflammatory Response After Burn and Inhalation Injury Is Mtor Dependent

**DOI:** 10.1093/jbcr/irac012.045

**Published:** 2022-03-23

**Authors:** Hannah R Hall, Cressida Mahung, Roland F Seim, Booker King, Shannon Wallet, Robert Maile

**Affiliations:** University of North Carolina at Chapel Hill, Chapel Hill, North Carolina; University of North Carolina at Chapel Hill, Washington, District of Columbia; University of North Carolina at Chapel Hill, Chapel Hill, North Carolina; UNC Jaycee Burn Center, Chapel Hill, North Carolina; University of North Carolina at Chapel Hill, Chapel Hill, North Carolina; UNC, Chapel Hill, North Carolina

## Abstract

**Introduction:**

Severe burn injury can lead to local and systemic activation of the innate immune system which can cause a dysfunctional and overt pro-inflammatory response, resulting in inflammatory complications and organ dysfunction. If there is an inhalation injury concomitant with burn, patients have a 3.6 times higher mortality rate and greater than 70% chance of developing secondary respiratory complications. Previous work has shown that the Mechanistic/ Mammalian Target of Rapamycin (mTOR) pathway is involved in signaling neutrophil activity in burn patients. Antagonists of mTOR stimulation represent possible immunomodulatory therapies. However, it is unclear if mTOR plays a role in the pulmonary distress seen after combined burn and smoke inhalation (B+I) injury. The goals of this study were to 1) characterize a novel mouse model of combined B+I injury and 2) investigate the role of mTOR in the pro-inflammatory response after B+I injury.

**Methods:**

We built upon our pre-established 20% total body surface area cutaneous burn mouse model by adding smoke inhalation injury and treating mice with rapamycin, a mTOR-specific inhibitor. In brief, mice were anesthetized before receiving controlled B+I, burn only, or sham injury. After resuscitation, they were given morphinated water for 24 hours before euthanasia and tissue collection. RNA and whole cells were extracted from lung and spleen tissue for analysis by Nanostring and Flow Cytometry, respectively. Fluid within the lung (bronchoalveolar lavage, BAL) and peripheral blood plasma were collected for pro-inflammatory cytokine quantification via magnetic bead multiplex assays.

**Results:**

Wildtype female C57BL/6 mice that underwent B+I injury exhibited elevated levels of protein, macrophages, and neutrophils in the lung cavity compared to burn alone. In addition, 29 genes were significantly differentially expressed in the lung tissue after B+I, suggesting that inhalation elicits a unique response when compared to burn alone. In the peripheral blood, B+I mice have a >4 fold increase in IL6 and 3 fold increase in MCP-1 pro-inflammatory cytokine levels. When we examined the role of mTOR in B+I injury, we found that pre-emptive rapamycin treatment leads to a reduction in peripheral blood pro-inflammatory cytokine levels, namely MCP1, TNFa, IL10, and IL2, and an increase in pro-inflammatory cytokine IL2 in the lung cavity. Rapamycin significantly affected expression levels of 46 genes in the lung and 36 genes in the spleen, indicating an mTOR-dependent response to inhalation injury.

**Conclusions:**

In conclusion, these data describe a valuable mouse model of B+I with inhalation-specific immune phenotypes and implicate mTOR in the inhalation-induced hyper pro-inflammatory response.

